# Anti-Infectious Agents against MRSA

**DOI:** 10.3390/molecules18010204

**Published:** 2012-12-24

**Authors:** Nobuhiro Koyama, Junji Inokoshi, Hiroshi Tomoda

**Affiliations:** Graduate School of Pharmaceutical Sciences, Kitasato University, 5-9-1 Shirokane, Minato-ku, Tokyo 108-8641, Japan; E-Mails: koyaman@pharm.kitasato-u.ac.jp (N.K.); inokoshij@pharm.kitasato-u.ac.jp (J.I.)

**Keywords:** MRSA, anti-infectious agents, peptidoglycan, teichoic acid, virulence factors

## Abstract

Clinically useful antibiotics, β-lactams and vancomycin, are known to inhibit bacterial cell wall peptidoglycan synthesis. Methicillin-resistant *Staphylococcus aureus* (MRSA) has a unique cell wall structure consisting of peptidoglycan and wall teichoic acid. In recent years, new anti-infectious agents (spirohexaline, tripropeptin C, DMPI, CDFI, cyslabdan, 1835F03, and BPH-652) targeting MRSA cell wall biosynthesis have been discovered using unique screening methods. These agents were found to inhibit important enzymes involved in cell wall biosynthesis such as undecaprenyl pyrophosphate (UPP) synthase, FemA, flippase, or UPP phosphatase. In this review, the discovery, the mechanism of action, and the future of these anti-infectious agents are described.

## 1. Introduction

**Figure 1:**
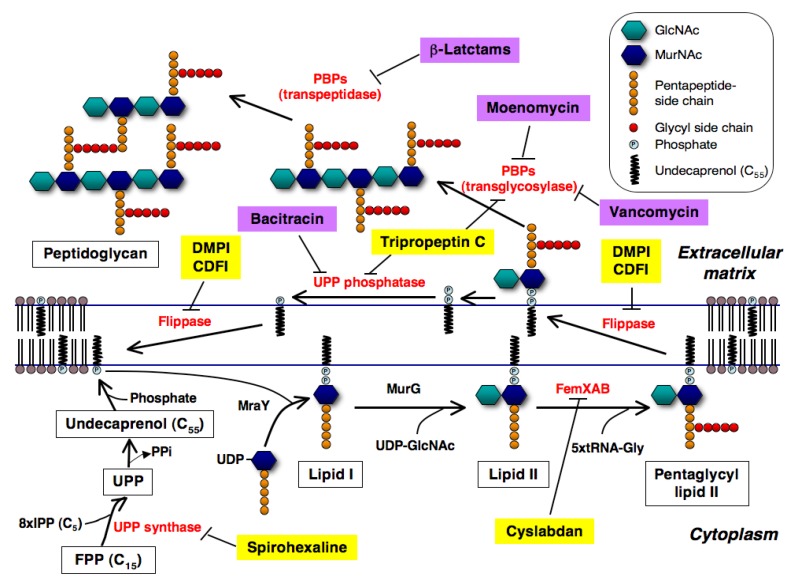
Biosynthetic pathway of peptidoglycan of *S. aureus* and new inhibitors involved in this pathway. New inhibitors and known antibiotics inhibiting this pathway are shown as yellow and purple highlights, respectively. Their inhibition sites are shown here.

Peptidoglycan, the major component of the bacterial cell wall, is an attractive target for the development of anti-infectious agents. It forms a giant macromolecule that surrounds the cell as a single, flexible meshwork and is intimately involved in cell division. The structure determines the cell shape and maintains cell integrity by protecting it against the high internal osmotic pressure. Important antibiotics including β-lactams and glycopeptides that target cell wall peptidoglycan synthesis have been clinically used [1]. In Figure 1, the biosynthetic pathway of peptidoglycan and the targets of well known antibiotics in peptidoglycan biosynthesis are illustrated. Peptidoglycan is composed of a series of short glycan chains of approximately 20 alternating *N*-acetylmuramic acid (MurNAc) and β-1-4-*N*-acetylglucosamine (GlcNAc) residues [2]. A pentapeptide is attached to each MurNAc as the stem peptide to form a murein monomer (MurNAc-GlcNAc-pentapeptide). In *Staphylococcus aureus*, the UDP-MurNAc-pentapetide is first transferred to a carrier lipid undecaprenyl phosphate (UP) in the cytoplasmic membrane by MraY to form lipid I, and then GlcNAc is added to it by MurG to form lipid II (Figure 1a). Next, a pentaglycine is extended from the L-Lys of the pentapeptide region in the MurNAc-GlcNAc-pentapetide by FemXAB to form pentaglycyl lipid II, which is then transferred outside through the membrane by flippase. After that, the transglycosylation and transpeptidation between a pentaglycyl murein monomer and an oligomeric peptidoglycan intermediate take place on the external surface of the membrane in a sequential reaction catalyzed by penicillin-binding proteins (PBPs); MurNAc of murein monomers is connected to GlcNAc by PBP transglycosylase to extend the glycan chains, and the terminal Gly of the pentaglycine of murein monomers is connected to the second D-Ala of the pentapeptide with the concomitant release of the terminal D-Ala by PBP transpeptidase to form crosslinks between murein monomers. β-Lactams inhibit PBP transpeptidase, resulting in the failure of peptidoglycan crosslinking; vancomycin and moenomcyin inhibit PBP transglycosylase, resulting in the failure of glycan chain formation [3,4,5], and bacitracin inhibits undecaprenyl pyrophosphate (UPP) phosphatase, resulting in the failure of the recycling of the carrier lipid UP [6]. Because bacterial cell wall peptidoglycan is not found in mammalian cells, these inhibitors show excellent selective toxicity.

Methicillin-resistant *S. aureus* (MRSA) is an important nosocomial and community-acquired pathogen that has also developed resistance to various antibiotics (β-lactams, quinolones, and aminoglycosides) [7]. MRSA infections cause a large number of deaths every year worldwide [8]. Vancomycin was considered to be the last-resort antibiotic for the treatment of MRSA infections, but MRSA resistance to vancomycin has been reported too [9,10]. This suggests that MRSA will likely acquire more resistance to vancomycin in the near future. Therefore, it is increasingly necessary to discover new antibiotics or to devise new measures that are effective against MRSA infections.

The concept of “anti-infectious drugs” includes not only compounds that inhibit the growth of pathogenic microorganisms statically or kill them (so called chemotherapeutics or antibiotics) and vaccines but also compounds that control microbial adaptation/survival or pathogenicity, potentiate the activities of known antibiotics, or enhance the host immune system against microbial infection. For example, β-lactamase inhibitors such as clavulanic acid, sulbactam, and tazobactam themselves show very weak or no antimicrobial (non-antibiotic) activity, but these compounds dramatically potentiate the antimicrobial activity of β-lactam antibiotics against β-lactamase-producing bacteria [11].

In recent years, anti-infectious compounds active against MRSA have been extensively searched for. Several compounds have been found to have new mechanisms of action against MRSA and are expected to be potential leads for the treatment of infection. They include microbial natural products like spirohexaline, tripropeptin C, and cyslabdan; and synthetic compounds such as DMPI, CDFI, 1835F03, targocil, and BPH-652. These compounds target peptidoglycan, wall teichoic acid, and a virulence factor of *S. aureus*. In this review, the discovery, biological activity, and mechanisms of action of these compounds are described.

## 2. New Inhibitors of Bacterial Cell Wall Peptidoglycan

### 2.1. Spirohexaline

#### 2.1.1. UPP Synthase as a Potential Target

UPP is a key lipid involved in the biosynthesis of peptidoglycan and other cell-wall polysaccharide components such as lipopolysaccharides, enterobacterial common antigen, capsule polysaccharides, and teichoic acids [12] (Figure 1). UPP-linked saccharides are also used for *N*-linked protein glycosylation that occurs in certain prokaryotes. In the cell wall synthetic pathway, UPP is needed for the synthesis and transport of hydrophilic GlcNAc-MurNAc-pentapeptides across the hydrophobic environment of the cytoplasmic membrane to the externally located sites of polymerization (Figure 1). UPP synthase catalyzes consecutive condensation reactions of farnesyl pyrophosphate with eight molecules of isopentenyl pyrophosphate to form UPP. This enzyme belongs to a group of *cis*-prenyltransferases that catalyze *cis*-double bonds during IPP condensation reactions. This enzyme is essential for bacterial cell growth and is not found in humans. Thus, UPP synthase is expected to be an attractive target for the development of anti-infectious agents that are effective against resistant bacteria, including MRSA and vancomycin-resistant *enterococci* (VRE).

#### 2.1.2. Screening of UPP Synthase Inhibitors

**Figure 2:**
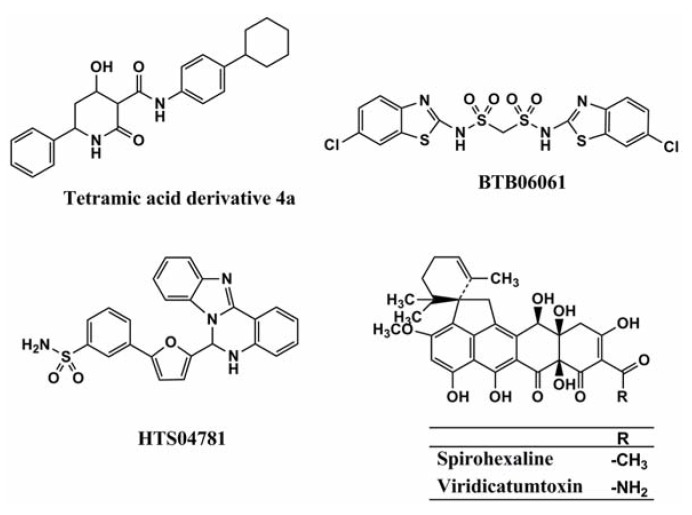
Structures of tetramic acid 4a, BTB06061, HTS04781, spirohexaline, and viridicatumtoxin.

For the reasons mentioned above, several groups have researched UPP synthase inhibitors. The GlaxoSmithKline group first discovered UPP synthase inhibitors by utilizing an enzyme-based assay system that measures Pi released in the enzymatic reaction of UPP synthase, and a cell-based assay system that analyzes the incorporation of [14C]isopentenylpyrophosphate (IPP). However, their active structures were undisclosed [13]. The Novartis group studied a pharmacophore model of a co-crystal structure of UPP synthase with its natural substrate in the active site of the enzyme, which successfully led to the discovery of tetramic acid derivative **4a** (Figure 2) as a potent inhibitor of *Staphylococcus pneumonia* UPP synthase [14]. Liang and coworkers also performed virtual screening based on the crystal structure of *Helicobacter pylori* UPP synthase, and discovered the sulfonyl bis-containing synthetic compound BTB06061 (Figure 2) as a potent and selective inhibitor of *H. pylori* UPP synthase [15]. Furthermore, Durrant *et al*. studied a docking model of substrates/inhibitors of UPP synthase based on the X-ray structures of UPP synthase-substrate (e.g., farnesyl diphosphate)/inhibitors (e.g., bisphosphonate drugs) complexes, leading to the development of the non-bisphosphonate synthetic compound HTS04781 that has potent inhibitory activity against *S. aureus* UPP synthase [16]. Recently, our group discovered a new compound named spirohexaline and the structurally related known viridicatumtoxin (Figure 2) as UPP synthase inhibitors from the culture broth of *Penicillium brasilianum* FKI-3368 [17]. These compounds have a hexacycline structure with a tetracyclic ring fused with a spiro-bicyclic ring. In our screening program, culture broths (samples) that showed antimicrobial activity against *S. aureus* and *Bacillus subtilis* were selected first. Then, such samples were evaluated by enzyme assays using recombinant *S. aureus* UPP synthase.

#### 2.1.3. Mechanism of Action of Spirohexaline

Table 1 summarizes the UPP synthase inhibitors reported to date. A number of UPP synthase inhibitors have been discovered with an enzyme-based or an *in silico* screening, but most of them do not show antimicrobial activity. Among them, tetramic acid derivative 4a, spirohexaline, and viridicatumtoxin show antimicrobial activity due to the inhibition of UPP synthase.

**Table 1:** UPP synthase inhibitors.

Compound	Structure classification	Source	MIC against MRSA	Inhibitory activity against UPP synthase **	Ref.
BTB06061	Bisphosphonate	Synthetic origin	N.R. *	71 M	[14]
Tetramic acid derivative 4a	Tetramic acid	Synthetic origin	N.R.	0.2 M	[15]
Spirohexaline	Hexacyclic ring	Fungus (*Penicillium brasilianum* FKI-3368)	6.25 g/mL	9.0 M	[16]
Viridicatumtoxin	Hexacyclic ring	Fungus (*Penicillium brasilianum* FKI-3368)	0.78 g/mL	4.0 M	[17]

* This is an abbreviation of no report; ** Inhibitory activity of the compounds against UPP synthase is expressed as an IC_50_ value.

Our group studied the mechanisms of action of spirohexaline [17]. We investigated the effect of spirohexaline and viridicatumtoxin on *E. coli* octaprenyl pyrophosphate (OPP) synthase which catalyzes the *trans*-type condensation reaction of FPP with IPP to generate C40 *trans*-octaprenyl pyrophosphate. Although spirohexaline and viridicatumtoxin inhibit UPP synthase activity with IC_50_ values of 9.0 and 4.0 μM, respectively, the compounds inhibit OPP synthase activity with IC_50_ values of 64.6 and 16.4 μM, respectively, indicating that they have higher selectivity towards *S. aureus* UPP synthase. Furthermore, we confirmed that these compounds inhibit UPP synthase activity using a [14C] IPP incorporation assay. As expected, spirohexaline and viridicatumtoxin more potently inhibit C55 production by UPP synthase than C40 and C80-90 production by OPP synthase and dehydrodolichyl-PP synthase. Spirohexaline and viridicatumtoxin show antimicrobial activity against Gram-positive bacteria, including clinically isolated MRSA. They appear to be ideal UPP synthase inhibitors because they show good correlation between the inhibition of UPP synthase and antibacterial activity.

Bacterial UPP synthase is recognized as a promising target for the development of new anti-infectious agents that are effective against resistant bacteria because it is essential in the biosynthesis of peptidoglycan and other cell-wall polysaccharide components. Therefore, spirohexaline is a candidate for the development of a new type of anti-infectious agents. Studying its *in vivo* efficacy is warranted.

### 2.2. Tripropeptin C

#### 2.2.1. Discovery

**Figure 3:**
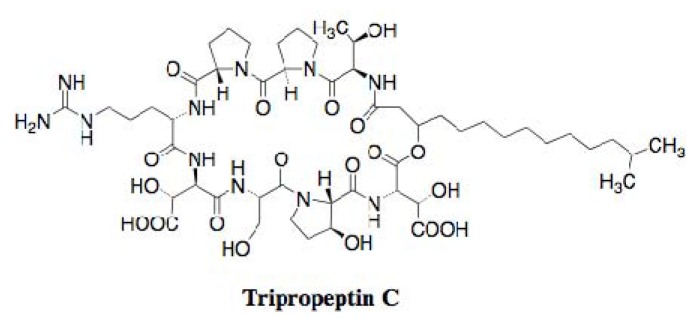
Structure of tripropeptin C.

Hashizume *et al*. screened microbial culture broths for new antibiotics effective against drug-resistant bacteria, including MRSA and vancomycin-resistant *enterococci* (VRE), which shows no cross-resistance with vancomycin and has low toxicity against mouse lymphocytic leukemia L-1210 cells. The screening results led to the discovery of tripropeptin C from the fermentation broth of the soil bacterium *Lysobacter* sp. strain BMK333-48F3 [18,19]. The compound is a new lipopeptide, consisting of a cyclic octapeptide core and a fatty acid side chain, 13-methyl-3-hydroxytetradecanoic acid (Figure 3).

#### 2.2.2. *In Vitro* and *in Vivo* Antimicrobial Activities

Tripropeptin C (Figure 3) shows potent inhibitory activity against Gram-positive bacteria, including MRSA, but is not active against Gram-negative bacteria, *Mycobacterium*, and *Candida*. The MIC values of tripropeptin C against several MRSA strains have been measured to be 0.78–1.0 µg/mL. The potency is almost the same as that of vancomycin. As expected, the compound is also very active against VRE with an MIC value of 4.0 µg/mL. Furthermore, tripropeptin C was found to act as a bactericidal agent against *S. aureus*, and to show no cross-resistance to available drugs, including vancomycin. The compound showed excellent therapeutic efficacy in a mouse septicemia model against MRSA and VRE with ED_50_ values of 2.52 and 20.0 mg/kg, respectively. The toxicity test in a mouse model indicated that the acute toxicity after intravenous administration was more than 300 mg/kg, and 14-day repetitious administration (100 mg/kg/d, i.v.) did not affect body weight and major organs, indicating that the compound has high safety in mammalian cells.

#### 2.2.3. Mechanism of Action

Hashizume *et al.* extensively studied the mechanism of action of tripropeptin C [20]. First, whole cell labeling experiments were carried out by investigating the incorporation of radioactive precursors ([^3^H]thymidine, [^3^H]uridine, [^3^H]leucine, and [^3^H]GlcNAc) into macromolecules using intact *S. aureus*. The compound selectively inhibited the incorporation of [^3^H]GlcNAc into macromolecules, suggesting that tripropeptin C blocked the synthesis of cell wall peptidoglycan. Furthermore, the accumulation of UDP-MurNAc-pentapeptide was observed in the cytoplasm of *S. aureus* when treated with tripropeptin C, indicating that tripropeptin C is not involved in the biosynthesis of UDP-MurNAc-pentapeptide. This suggested that the inhibition site lies within one of the subsequent membrane-associated steps in peptidoglycan biosynthesis. Next, they investigated the effect of cell wall-related materials on antimicrobial activity by tripropeptin C. The antimicrobial activity of tripropeptin C was weakened in the presence of prenyl pyrophophates, including UPP. On the other hand, the antimicrobial activity of tripropeptin C was unaffected by the presence of sodium pyrophosphate or UDP-containing pyro-phosphate. Direct binding of tripropeptin C to UPP was observed by analyses of mass spectrometry and thin-layer chromatography, suggesting that tripropeptin C inhibits UPP phosphatase activity, which is involved in the lipid cycle of peptidoglycan synthesis. Finally, they demonstrated that tripropeptin C inhibited the phosphatase activity of *Micrococcus luteus in vitro* as well as bacitracin, a known inhibitor of the UPP phosphatase. Furthermore, the effect of tripropeptin C on the accumulation of peptidoglycan precursors and lipid carriers through the mevalonate pathway was studied by a [^14^C]mevalonolactone incorporation assay. As expected, tripropeptin C caused the intracellular accumulation of UPP, which was consistent with the results of the phosphatase assay. Interestingly, the accumulation of a glycine-containing lipid intermediate was also observed with longer exposure to the compound, similar to vancomycin. Thus, tripropeptin C might block the transglycosylation step or flippase activity together with the inhibition of dephosphorylation of UPP.

#### 2.2.4. Cyclic Peptide Antibiotics Binding to Peptidoglycan Biosynthetic Molecules

**Figure 4:**
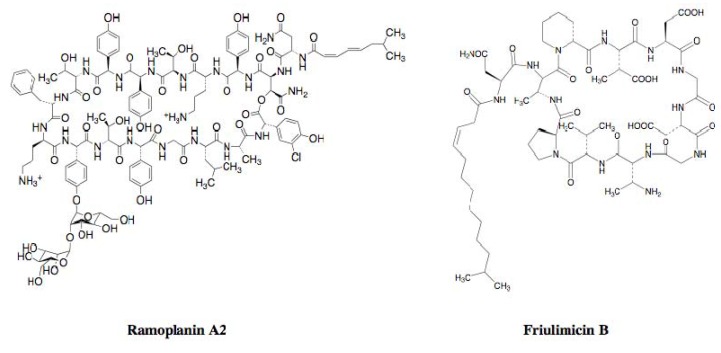
Structures of ramoplanin A2 and friulimicin B.

Cyclic peptide antibiotics have recently been reported to bind to peptidoglycan biosynthetic molecules as well as tripropeptin C. Ramoplanin A2 (Figure 4), an anti-infectious agent against antibiotic-resistant Gram-positive pathogens, was originally isolated from the culture broth of an actinomycete strain, *Actinoplanes* sp. ATCC 33076. The compound is a glycopeptide composed of 17 amino acid residues condensed with an *N*-acylated fatty acid tail and a sugar moiety, which binds to both lipid I and lipid II, resulting in inhibition of the late-stage enzymes involved in peptidoglycan biosynthesis, MurG and PBP transglycosylase [21,22]. Friulimicin B (Figure 4), discovered as a new metabolite from the actinomycete strain *Actinoplanes friuliensis* HAG 010964, possesses a lipopeptide core structure composed of 11 amino acid residues and an *N*-acylated fatty acid [23]. The compound binds to UP, resulting in the blockade of all biosynthetic pathways that make use of undecaprenyl phosphate, such as peptidoglycan, wall teichoic acid, and polysaccharide capsule biosyntheses [24].

### 2.3. DMPI, CDFI, and Cyslabdan

#### 2.3.1. Screening for β-Lactam Potentiators against MRSA

Several groups have searched for anti-infectious drugs that potentiate the antimicrobial activity of clinically used β-lactams. A large number of screenings for β-lactam potentiators against MRSA have been reported [25,26,27,28,29,30,31,32,33,34,35,36]. The screening systems are all based on a common technique, namely, comparing the anti-MRSA activity of samples in the presence and absence of β-lactams, and selecting samples that show more potent anti-MRSA activity in the presence of β-lactams.

#### 2.3.2. Potentiators of β-Lactam Activity against MRSA

**Figure 5:**
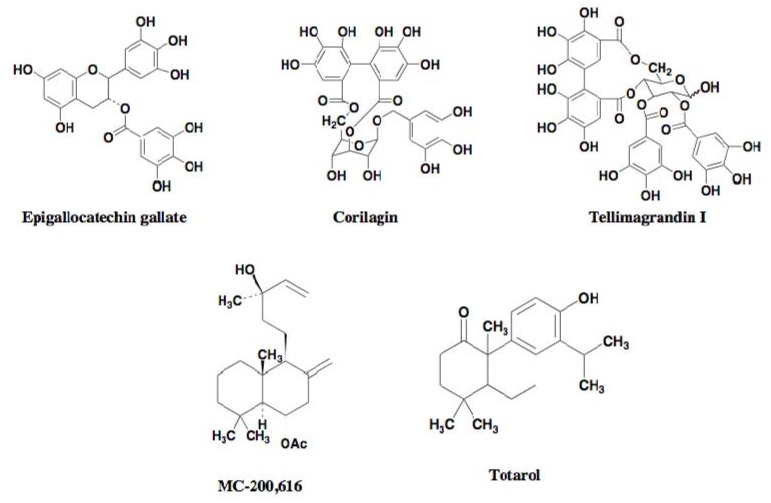
Structures of known β-lactam potentiators against MRSA.

Using the above screening systems, several groups have discovered potentiators of β-lactams against MRSA. In 1995, the Microcide group first discovered the synthetic diterpene MC-200,616 (Figure 5) [25]. In 1999, Paul *et al.* reported the diterpene totarol (Figure 5) isolated from the totara tree [26]. In 2000–2001, Tsuchiya *et al*. reported polyphenols from plants such as corilagin from *Arctostaphylos uva-urs*, tellimagrandin I from the *Rosa canina* L., and epigallocatechin gallate isolated from tea [27,28,29] (Figure 5).

**Figure 6:**
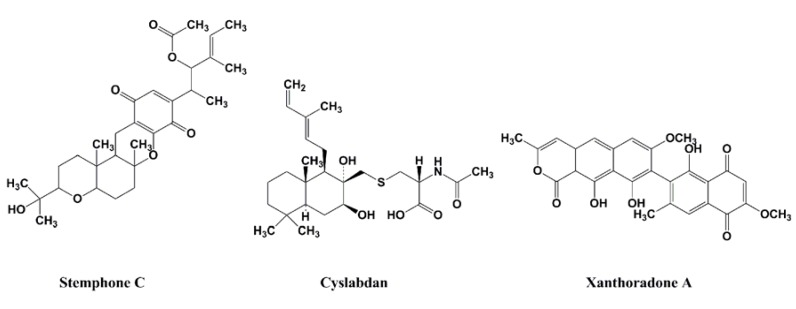
Structures of stemphone C, cyslabdan, and xanthoradone A.

In 2005, our group also discovered stemphones [30,31], followed by cyslabdan [32,33,34] and xanthoradones [35,36], from microbial sources (Figure 6). Stemphones, new metabolites produced by *Aspergillus* sp. FKI-2136, have a tetracyclic quinone core structure. Cyslabdan, a new metabolite produced by *Streptomyces* sp. K04-0144, has a labdan-type diterpene core structure connected with *N*-acetylcysteine via a thioether linkage. Xanthoradones, new metabolites produced by *Penicillium radicum* FKI-3765-2, have an aromatic ring-containing heterodimer core structure.

**Figure 7:**
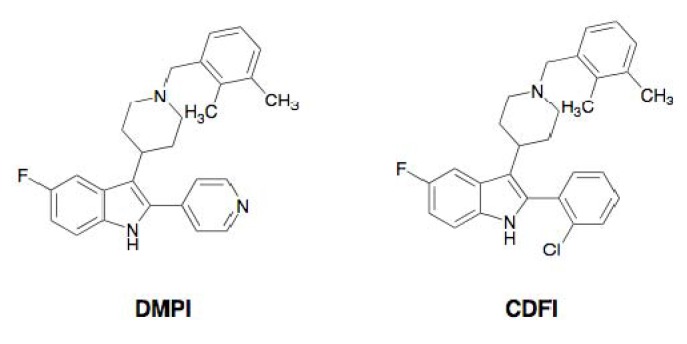
Structures of DMPI and CDFI.

Recently, the Merck group discovered the synthetic compounds DMPI and CDFI from high-throughput screening [37]. Both synthetic compounds possess an indole skeleton fused with a dimethylbenzylpiperidine (Figure 7).

#### 2.3.3. Activity of β-Lactam Potentiators against MRSA

The activity of β-lactam potentiators is summarized in Table 2, in which the MIC values of β-lactams against MRSA are compared in the presence or absence of potentiators.

**Table 2:** Activity of β-lactam potentiators against MRSA.

Compound	Structure classification	Source	Potentiating activity of β-lactam *				Ref.
			β-Lactam	MIC (µg/mL)		Potentiation ratio (fold) **	
				None	+Compound		
MC-200,616	Diterpene	Synthetic origin	Imipenem	32	0.03	1067	[25]
Totarol	Diterpene	Plant (totara tree)	Methicillin	1024	4	256	[26]
Epigallocatechin gallate	Polyphenol	Plant (tea)	Imipenem	128	0.5	256	[27]
Corilagin	Polyphenol	Plant (*Arctostaphylos uva-urs*)	Imipenem	64	0.03	2133	[28]
Tellimagrandin I	Polyphenol	Plant (*Rosa canina* L.	Oxacillin	512	1	512	[29]
Stemphone C	Tetracyclic quinone	Fungus (*Aspergillus* sp. FKI-2136)	Imipenem	16	0.03	533	[30,31]
Cyslabdan	Diterpene	Actinomycete (*Streptomyces* sp. K04-0144)	Imipenem	16	0.015	1067	[32,33,34]
Xanthoradone A	An aromatic ring-containing heterodimer	Fungus (*P.radicum* FKI-3765-2)	Imipenem	16	0.03	533	[35,36]
DMPI	Indole	Synthetic origin	Imipenem	32	2	16	[37]
CDFI	Indole	Synthetic origin	Imipenem	32	2	16	[37]

* Potentiating activity of β-lactams against MRSA is evaluated by measuring the MIC value of β-lactams against MRSA in the absence and presence of the compounds (1/4 MIC). Name of β-lactams used for the evaluation is shown here. ** Potentiation ratio is expressed as the MIC value in the absence of the compounds/the MIC value in the presence of the compounds.

All of the potentiators enhance β-lactam activity against MRSA, while most of the potentiators themselves have weak activity against MRSA. But under the experimental conditions, the concentration of potentiators was set up at one-fourth of the MIC value, which shows no effect on the growth of MRSA. Among them, stemphones, cyslabdan, xanthoradones, epigallocatechin gallate, corilagin, tellimagrandin I, MC-200,616, and totarol were found to be very potent potentiators, yielding 256- to 2133-fold potentiation of the β-lactam activity. DMPI and CDFI were moderate potentiators, enhancing the imipenem activity against MRSA by 16-fold (Table 2). Stemphones, cyslabdan, corilagin, and tellimagrandin I had no potentiating activity on other antibiotics such as macrolides, aminoglycosides, tetracyclines, and quinolones.

It is well known that MRSA possesses a β-lactam-insensitive transpeptidase named PBP2’ or PBP2a as a resistant mechanism. Several groups have extensively studied the mechanism of action of certain potentiators, indicating that epigallocatechin gallate, corilagin, tellimagrandin I, and MC-200,616 inhibit PBP2’ activity [27,38], and that totarol suppresses the expression of PBP2’ [26].

#### 2.3.4. Mechanism of Action of DMPI and CDFI

The Merck group studied the mechanism of action of DMPI and CDFI [37]. From whole cell labeling experiments using radioactive precursors ([^3^H]thymidine, [^3^H]uridine, [^3^H]leucine, [^14^C]glycine, and [^3^H]glycerol), the compounds selectively inhibited the incorporation of [^14^C]glycine into macromolecules in *S. aureus*, suggesting that they blocked the synthesis of cell wall peptidoglycan. To further study the inhibition step of the compounds in cell wall peptidoglycan biosynthesis, a recently developed antisense RNA technique was utilized. This technique was based on the conditional expression of essential genes of *S. aureus* by the induction of antisense RNA under the control of a xylose-inducible promoter. Reduction of essential genes is known to weaken cell growth and leads to increased sensitivity to the compounds in these strains since the levels of the target mRNA and thus target protein proportionally decrease. This technique can be used to predict the mechanism of action of a compound.

Using this method, the growth inhibition caused by DMPI and CDFI was compared between control *S. aureus* strain and various antisense RNA-induced *S. aureus* strains. As a result, a single hypersensitive strain, in which *sav1754* antisense RNA was induced, was identified as a prominent and reproducible candidate. Furthermore, this antisense RNA-induced *S. aureus* was cultured in the presence of high concentrations of xylose, resulting in complete growth inhibition. This result implied that SAV1754 is an essential gene in *S. aureus*. Furthermore, SAV1754-overexpressing *S. aureus* reversely led to decreased sensitivity of the strain to the compounds. These results indicated that the mechanism of action of DMPI and CDFI was involved in the product of the *SAV1754* gene. Furthermore, genetic analysis of *S. aureus* mutants resistant against these compounds revealed that they have a distinct point mutation (I18M, P44Q, or P257S) in the *SAV1754* ORF region of the control strain. Allelic copies of SAV1754 containing these mutations were introduced by homologous recombination to investigate whether they affect the antimicrobial activity of DMPI. Each of these mutants was sufficiently resistant to both DMPI and CDFI. These results demonstrated that the molecular target of DMPI and CDFI is SAV1754.

The same group further studied the effect of genetic inactivation of SAV1754 on the imipenem potentiating activity of DMPI and CDFI against MRSA. In *sav1754* antisense RNA-induced MRSA, the sensitivity of imipenem against MRSA was found to markedly increase. Furthermore, this MRSA strain resulted in profound hypersensitivity to other β-lactams tested, including ertapenem, cefepime, ceftazidime, and ceftriaxone. This result is highly consistent with the hypersensitivity of MRSA to β-lactams in combination with DMPI and CDFI. They concluded that DMPI and CDFI inhibit SAV1754, thereby restoring MRSA susceptibility to β-lactams.

SAV1754 is conserved among Gram-positive bacteria, but its function has not been characterized. The analogous protein MurJ in *S. aureus*, named flippase, is ubiquitously conserved among Gram-negative bacteria, and works to translocate lipid II from the cytoplasmic region to the periplasmic surface of the cell membrane. Based on these findings, the researchers speculated that SAV1754 performs a similar flippase function in cell wall biosynthesis. Inhibitors of SAV1754 had not been previously reported, and DMPI and CDFI are potential leads for enhancing β-lactams that are ineffective against MRSA.

#### 2.3.5. Mechanism of Action of Cyslabdan

**Figure 8:**
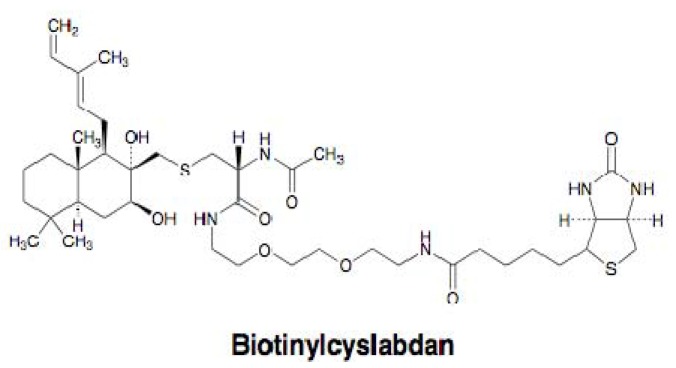
Structure of biotinylcyslabdan.

Our group studied the mechanism of action of cyslabdan by a biochemical and proteomics approach [39]. As shown in Figure 6, cyslabdan has a labdan-type structure similar to totarol and MC-200,616, whose mechanism of action is reported to involve PBP2’. However, cyslabdan has no effect on the expression and activity of PBP2’. Furthermore, cyslabdan does not inhibit serine β-lactamases, and has a mechanism of action against MRSA different from the other labdan compounds. Therefore, a distinct strategy was taken to uncover the mechanism: Biotinylcyslabdan (Figure 8) was prepared by modifying the carboxyl group in the *N*-acetylcysteine part, which retained the potentiating activity of imipenem against MRSA. Using this probe, an analysis of cyslabdan-binding proteins resulted in the detection of one protein band of 50 kDa, which was identified as SAR1388 by LC-MS/MS. We further found that cyslabdan has affinity for recombinant SAR1388 protein.

SAR1388, named FemA, is a factor essential for the expression of methicillin resistance , which is involved in peptidoglycan biosynthesis in MRSA. *S. aureus* has a unique pentaglycine interpeptide bridge between murein monomers in peptidoglycan [40]. Three enzymes are involved in interpeptide bridge formation in *S. aureus*: FemX works to add the first Gly from *L*-Lys of the murein monomer, FemA adds the second and third Gly, and FemB adds the forth and fifth Gly. A FemA mutant has been reported to restore susceptibility to β-lactams against MRSA [41,42]. These findings suggest that the molecular target of cyslabdan is FemA. We found that cyslabdan did indeed inhibit the enzymatic activity of FemA, which catalyzes the conversion of monoglycyl lipid II to triglycyl lipid II. Furthermore, cyslabdan-treated MRSA was found to accumulate monoglycyl and nonglycyl murein monomers. A FemA mutant of MRSA has been reported to accumulate the same mureine monomers in the MRSA peptidoglycan, which is very similar to our results [42]. Taken together, we concluded that cyslabdan primarily targets FemA, thereby inhibiting the synthesis of the pentaglycine interpeptide bridge.

**Figure 9:**
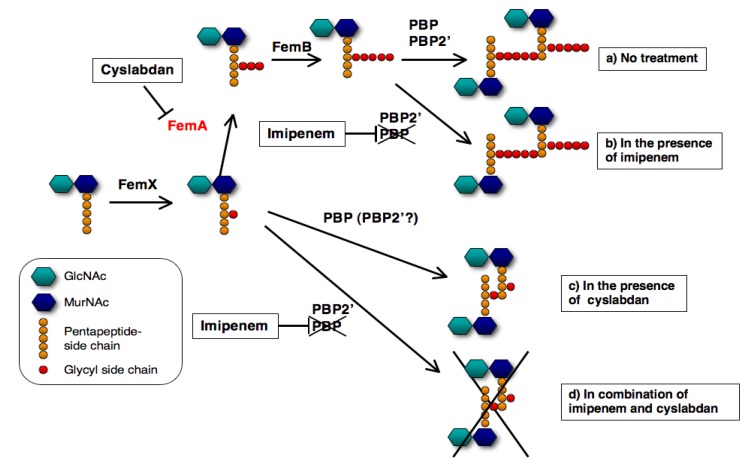
Proposed mechanism underlying the effect of cyslabdan on the activity of imipenem against MRSA. The following four cases are shown here: (**a**) no treatment (**b**) in the presence of imipenem, (**c**) in the presence of cyslabdan and (**d**) combination of imipenem and cyslabdan.

A proposed mechanism of the synergic action of cyslabdan with imipenem against MRSA is illustrated in Figure 9. When MRSA is treated with cyslabdan, FemA is inhibited resulting in the accumulation of monoglycyl or nonglycyl murein monomers. However, cyslabdan itself shows almost no effect on growth of MRSA, meaning that PBP and/or PBP2’ can recognize monoglycyl murein monomers as a substrate and crosslink between them to build up peptidoglycan. On the other hand, in combination with imipenem and cyslabdan, the growth of MRSA is completely inhibited, indicating that imipenem-insensitive PBP2’ cannot crosslink between monoglycyl mureins, leading to failure of MRSA peptidoglycan formation. Taken together, the biosynthetic pathway for the pentaglycine interpeptide bridge is a potential target for restoring the potency of drugs that are ineffective against MRSA. Such inhibitors will be promising candidates for combination therapies to combat MRSA.

## 3. Inhibitors of Wall Teichoic Acid

### 3.1. Wall Teichoic Acid as a Target of Anti-Infectious Agents against S. aureus

**Figure 10:**
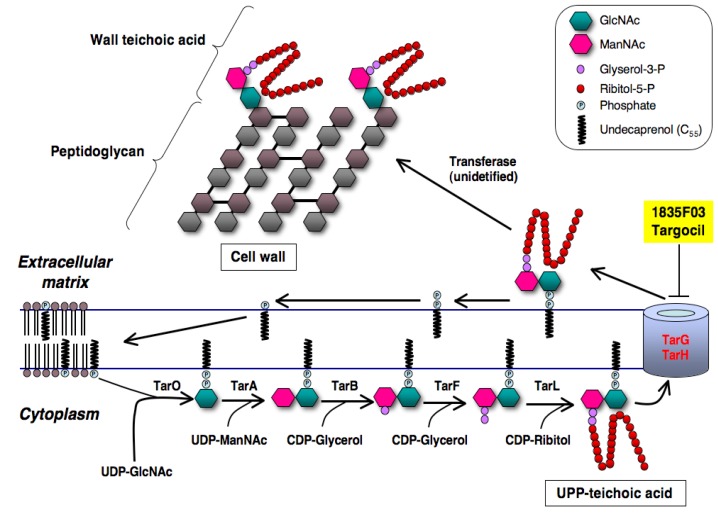
Biosynthetic pathway of wall teichoic acid of *S. aureus*. The inhibition site of new inhibitors (1835F03 and targocil) is shown here.

*S. aureus* has a unique wall teichoic acid (WTA) on the surface of the cell that consists of anionic polymers of poly(ribitol-phosphate) and disaccharide in which the anomeric hydroxyl group is covalently attached to the MurNAc of peptidoglycan *via* the phosphate group. Genetic analysis has revealed the WTA biosynthetic pathway in *S. aureus* [43,44] (Figure 10): TarO first works to transfer a UDP-GlcNAc to the carrier lipid UP in the cytoplasmic membrane, sharing a mechanism similar to peptidoglycan biosynthesis. Then, TarA adds an *N*-acetyl-D-mannosamine (ManNAc) to a UDP-GlcNAc to form a UPP-disaccharide. Next, TarB and TarF add two to three glycerol 3-phosphate units to the terminal ManNAc moiety of the UPP-disaccharide. Finally, TarL expands the poly(ribitol-phosphate) from the terminal glycerol 3-phosphate moiety to form the UPP-teichoic acid. A complex of two-component transporters TarG and TarH catalyzes the export of the UPP-teichoic acid outside through the membrane, and then an unidentified transferase transfers the UPP-teichoic acid to the peptidoglycan with concomitant release of a UPP to form WTA. WTA biosynthesis is expected to be a potential target for the development of new anti-infectious agents against MRSA given that the deletion of biosynthetic genes of WTA attenuates *S. aureus* infection in animal models [44].

### 3.2. Discovery of 1835F03

**Figure 11:**
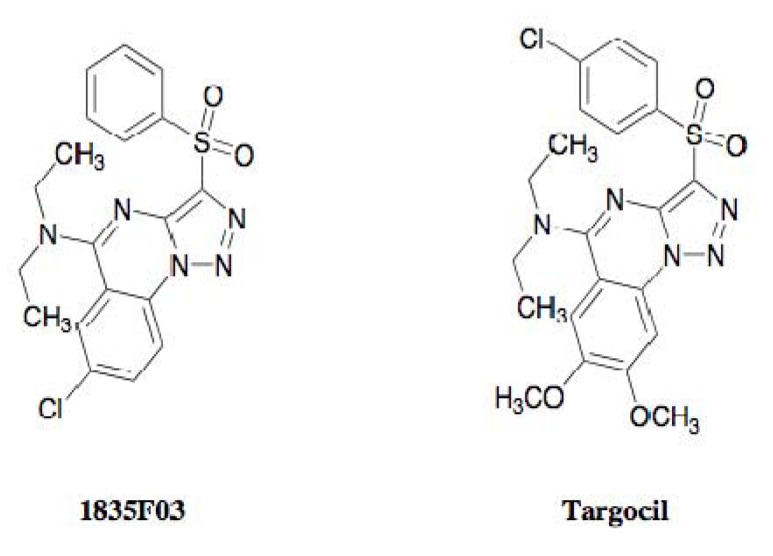
Structures of 1835F03 and targocil.

Walker and coworkers found that *tarO* and/or *tarA*-deficient *S. aureus* mutants are able to grow even though downstream genes such as *tarBFGHL* are deleted, whereas *S. aureus* harboring intact WTA biosynthetic genes are not able to grow when the function of the gene product is inhibited [45] (Figure 10). They applied these findings to establish an original assay system, and screened samples that showed selective antimicrobial activity against wild-type *S. aureus* but not against *tarO*-deficient *S. aureus* mutants. In 2009, they discovered the synthetic compound 1835F03 from a chemical library during this screening program [45]. The compound possesses a quinoline skeleton fused with a 1,2,3-triazole (Figure 11).

### 3.3. Molecular Target of 1835F03

The mechanism of action of 1835F03 has been extensively studied with biochemical and genetic analyses. As expected from the screening method, 1835F03 selectively inhibits the growth of wild-type *S. aureus* with an MIC value of 1.3 μg/mL, but not the *tarO*-deficient mutant. The compound is predicted to target a step later than TarA in WTA biosynthesis [45]. The compound is very active against clinically isolated MRSA strains whereas it is not active against other Gram-positive bacteria, including *Streptococcus penumoniae* and *B. subtilis*. To further investigate the mechanism of action of 1835F03, *S. aureus* expressing *tarO* under the control of an isopropyl β-D-1-thiogalactopyranoside (IPTG)-inducible promoter was constructed. When TarO was not expressed in the absence of IPTG, 1835F03 had no effect on the growth of *S. aureus*; in its presence, the compound markedly inhibited the growth. This result indicated that the molecular target of 1835F03 is involved in the WTA biosynthetic pathway, and that the compound acts on one of the conditionally essential WTA biosynthetic enzymes except for TarO and TarA. Next, Walker’s group showed that the compound had no effect on cytoplasmic proteins such as TarB, TarF, and TarL in biochemical assays, suggesting that 1835F03 targets a subsequent step in the WTA biosynthetic pathway (Figure 10). Furthermore, overexpression of TarG and TarH was sufficient to weaken the antimicrobial activity of 1835F03 against *S. aureus*, implying that TarG and TarH are the molecular targets. The gene products are reported to form a transporter complex [45,46]. To identify which proteins the compound acts on, 1835F03-resistant mutants were generated. Among five mutants obtained, two WTA-producing mutants were selected by utilizing the binding affinity of *S. aureus* bacteriophage for WTA because the mutants that were not able to biosynthesize WTA would have mutations in *tarO* and *tarA*. Analysis of the *tar* genes from two mutants revealed that they contain point mutations such as F82L and W73C in *tarG*. These results indicated that the molecular target of 1835F03 is TarG.

### 3.4. Development of Targocil as an Improved WTA Inhibitor

In 2010, the same group further studied the lead optimization of 1835F03 through a structure-activity relationship study, successfully leading to the development of a new analog named targocil [47] (Figure 11). Targocil has a 2,3-dimethoxy substitution in the quinoline moiety and has a 4-chloro substitution at its terminal benzene, which is slightly different from the chemical structure of 1835F03. The compound showed antimicrobial activity against *S. aureus* with an MIC value of 0.3 µM, which is 12 times as strong as the parent compound. Also, targocil is active against MRSA with the same potency. The *in vivo* study indicated that targocil caused no adverse effects with a dose of 75 mg/kg in a mouse model. Furthermore, target analysis with targocil-resistant mutants confirmed that targocil shares the same target TarG as 1835F03. In current, targocil is evaluated in pre-clinical trial for treatment of MRSA infections by the research team of Harvard University.

## 4. Inhibitors of a Virulence Factor

### 4.1. The Virulence Factor Staphyloxanthin as a Target of Anti-Infectious Agents against S. aureus

**Figure 12:**
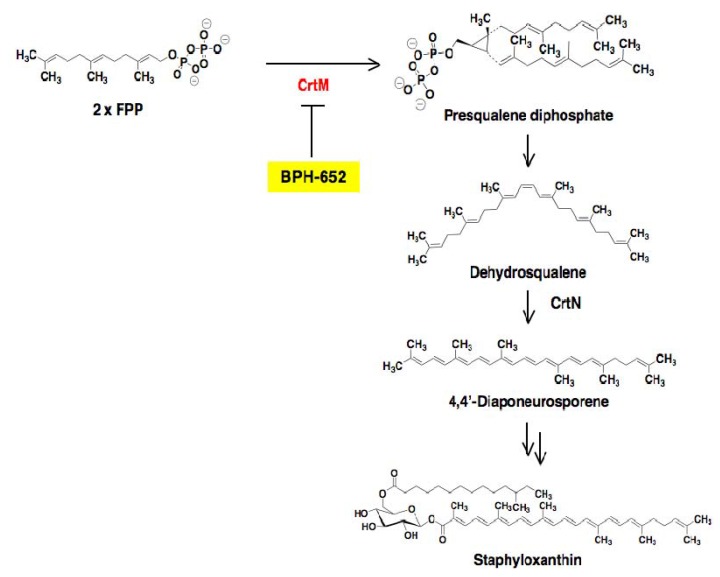
Biosynthetic pathway of the virulence factor staphyloxanthin. In *S. aureus,* the biosynthetic pathway involves initial formation of presqualene diphosphate from 2 × FPP, which is catalyzed by CrtM. After that, dehydrosqualene and 4,4'-diaponeurosporene are biosynthesized in turn. Finally, the formation of staphyloxanthin is completed *via* several steps from 4,4'-diaponeurosporene. The inhibition site of BPH-652 is shown here.

The yellow pigment of *S. aureus*, staphyloxanthin, is a virulence factor essential for the infection of hosts, and is involved in the resistance to reactive oxygen species and host neutrophil-based killing [48,49]. The biosynthetic pathway of staphyloxanthin is shown in Figure 12. Genetic analysis has revealed that the deletion of the early biosynthetic enzyme dehydrosqualene synthase (CrtM) has no effect on the growth of *S. aureus*, but results in marked attenuation of the virulence of *S. aureus* in a mouse model [49]. Therefore, the staphyloxanthin biosynthetic pathway is a potential target for the development of new anti-infectious agents against MRSA by weakening its virulence.

### 4.2. Screening for Inhibitors of Staphyloxanthin Biosynthesis in S. aureus

Inhibitors of staphyloxanthin biosynthesis reported to date are summarized in Table 3. In 2008, Oldfield and coworkers reported the synthetic compound BPH-652 as the first inhibitor of staphyloxanthin biosynthesis [50]. Based on bioinformatics findings that a key enzyme, CrtM, of stanphyloxanthin biosynthesis has high structural similarity to human squalene synthase (SQS), known SQS inhibitors were re-evaluated in an enzyme-based assay using recombinant *S. aureus* CrtM.

**Table 3:** Inhibitors of staphyloxanthin biosynthesis in *S. aureus*.

Compound	Structure classification	Source	Ref.
BPH-652	Biphenyl ether	Synthetic origin	[50]
Cerulenin	Epoxy fatty acid	Fungus (*Cephalosporium caerulens* KF-140)	[51]
Dihydrobisvertinol	Dibenzofuran	Fungus (*Verticillium intertextum*)	[51]
Xanthohumol	Chalcone	Hops plant	[51]
Zaragozic acid	Bicyclo ring	Fungus (*Phoma* sp.)	[51]
6-Deoxy-8-*O*-methylrabelomycin	Anthraquinone	Actinomycete (*Streptomyces badius* 4-6)	[51]
Tetrangomycin	Anthraquinone	Actinomycete (*Streptomyces badius* 4-6)	[51]

**Figure 13:**
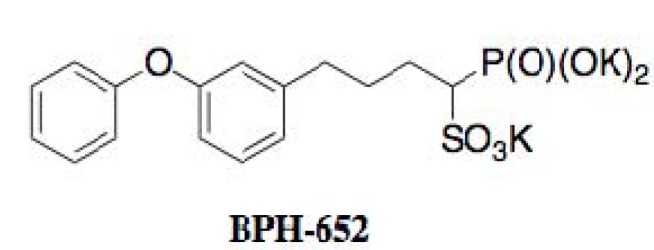
Structure of BPH-652.

**Figure 14:**
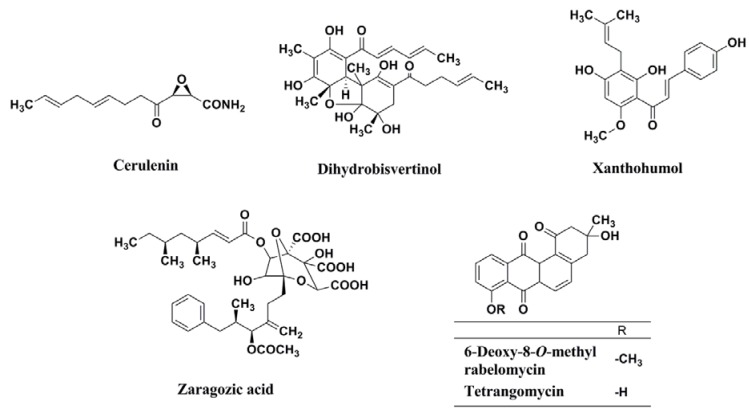
Structures of cerulenin, dihydrobisvertinol, xanthohumol, zaragozic acid, 6-deoxy-8-*O*-methylrabelomycin, and tetrangomycin.

The result led to the discovery of BPH-652 that possesses a biphenyl skeleton containing a butyryl phosphosulfonate (Figure 13). Very recently, our group also established a convenient colorimetric assay system using MRSA, and discovered four known inhibitors of lipid metabolism, cerulenin (an inhibitor of fatty acid synthase), dihydrobisvertinol, xanthohumol (an inhibitor of diacylglycerol acyltransferase), and zaragozic acid (an inhibitor of squalene synthase) from a natural product library, and discovered two known anthraquinones, 6-deoxy-8-*O*-methylrabelomycin and tetrangomycin, from actinomycete culture broths [51]. As shown in Figure 14, these compounds have a core structure different from the biphenyl ether BPH-652.

### 4.3. Biological Activity of BPH-652

Oldfield and coworkers extensively studied the biological activity of BPH-652 [50]. BPH-652 inhibited CrtM of *S. aureus* with a *K*_i_ value of 1.5 nM. Correspondingly, the pigmentation of *S. aureus* was suppressed with IC_50_ values of 100 to 300 nM. As expected from previous genetic deletion experiments of CrtM [49], BPH-652 did not directly inhibit the growth of *S. aureus*. Furthermore, BPH-652-treated *S. aureus* became 15-fold increased susceptibility by hydrogen peroxide and 4-fold decreased survival ability in whole blood cells, suggesting that the yellow pigment is involved in self-defense as an antioxidant. Finally, the *in vivo* efficacy of BPH-652 on *S. aureus* infection was confirmed in two mouse models of nasal colonization and intraperitoneal infection. BPH-652 treatment decreased the survival of *S. aureus* in the kidney by 98%, revealing that BPH-652 showed very excellent therapeutic efficacy without toxicity. Notably, BPH-652 was originally studied in early clinical trials as a cholesterol-lowering agent, and proved to have its low toxicity profile because the compound had no effect on the growth of various human cell lines. BPH-652 is a non-antibiotic compound, but elicits anti-MRSA activity by inhibiting the yellow pigmentation, which plays an important role in self-defense of MRSA from the host. This compound is expected to be a new type of anti-infectious agent against MRSA.

## 5. Conclusions

New anti-infectious compounds active against MRSA that have recently been discovered based on unique screening ideas are reviewed in this article. Spirohexaline of fungal origin and synthetic BPH-652 were discovered in enzyme-based assays for UPP synthase and CrtM, respectively, and were found to show anti-MRSA activity. Tripropeptin C of bacterial origin, synthetic DMPI, synthetic CDFI, cyslabdan of actinomycete origin, and synthetic 1835F03 were discovered in cell-based assays. Tripropeptin C inhibits UPP phosphatase and transglycosylase, DMPI and CDFI inhibit flippase, cyslabdan inhibits FemA, and 1835F03 inhibits TarG. Their molecular targets were elucidated by not only biochemical methods but also by knockdown of proteins using antisense RNA and identification of binding proteins. Thus, these anti-infectious agents target enzymes involved in the biosynthesis of MRSA specific cell wall peptidoglycan and WTA, most of which are different from those of clinically used antibiotics.

Information about these anti-infectious compounds includes new structural features and new mechanism of actions. Since MRSA infection is getting difficult to be cured by typical antibiotics, the compounds are expected to be potential leads for the development of a new type of anti-infectious agents active against MRSA. Particularly, tripropeptin C, targocil and BPH-652 prove active *in vivo* using mouse models. These agents are expected to be promising leads to combat MRSA. Furthermore, these compounds will serve as chemical tools for investigating the molecular mechanism on the cell wall biosynthesis and the virulence factor of MRSA.
